# A retrospective analysis of clinicopathological and prognostic characteristics of ovarian tumors in the State of Espírito Santo, Brazil

**DOI:** 10.1186/1757-2215-4-14

**Published:** 2011-08-09

**Authors:** Marcela F Paes, Renata D Daltoé, Klesia P Madeira, Lucas CD Rezende, Gabriela M Sirtoli, Alice L Herlinger, Leticia S Souza, Luciana B Coitinho, Débora Silva, Murilo F Cerri, Ana Cristina N Chiaradia, Alex A Carvalho, Ian V Silva, Leticia BA Rangel

**Affiliations:** 1 Laboratório de Biologia Celular e Molecular do Câncer Humano, Departamento de Ciências Farmacêuticas, 2° Andar, Sala 08, Centro de Ciências da Saúde, Universidade Federal do Espírito Santo, Maruípe, Vitória, ES - Brazil. CEP: 29043-900; 2 Centro de Ciências Agrárias, Universidade Federal do Espírito Santo, Alto Universitário, s/n° - Cx Postal 16, Guararema, Alegre, ES -Brazil. CEP: 29500-000; 3 Laboratório de Biologia Celular do Envelhecimento, Departmaneto de Morfologia, 1° Andar, Sala 05, Centro de Ciências da Saúde, Universidade Federal do Espírito Santo, Maruípe, Vitória, ES - Brazil; 4 Departamento de Patologia, Hospital Cassiano Antônio de Moraes (HUCAM), Avenida Marechal Campos, s/n°, Maruípe, Vitória, ES - Brazil. CEP: 29040-191

**Keywords:** ovarian neoplasias, Espírito Santo, retrospective study, clinical outcome, gynecological disease

## Abstract

**Background:**

Ovarian cancer is sixth most common cancer among women and the leading cause of death in women with gynecological malignancies. Despite the great impact ovarian cancer has on women's health and its great impact in public economy, Brazil still lacks valuable information concerning epidemiological aspects of this disease

**Methods:**

We've compiled clinical data of all ovarian tumors registered at the two public hospitals of reference (1997 - 2007), such as: patients' age at diagnosis, tumor histological type, tumor stage, chemotherapy regimens, chemotherapy responsiveness, disease-free survival, and overall survival.

**Results:**

Women's mean age at diagnosis was 54.67 ± 13.84 for ovarian cancer, 46.15 ± 11.15 for borderline tumors, and 42.01 ± 15.06 for adenomas. Among epithelial ovarian cancer cases, 30.1% were of serous, 13.7% were of mucinous, and 13.7% were of endometrioid type; exceptionally serous carcinoma was diagnosed in women younger than 30 years old. Endometrioid cancer had lower disease-free survival than others (p < 0.05). Cases were predominantly diagnosed as poor prognosis disease (FIGO III and IV, 56.2%). Regarding responsiveness to platinum-based therapy, 17.1% of patients were resistant, whereas 24.6%, susceptible. From these, we found equally responsiveness to platinum alone or its association with paclitaxel or cyclophosphamide.

**Discussion:**

Our data agreed with other studies regarding mean patients' age at diagnosis, histological type frequency, FIGO stages distribution, and chemotherapy regimens. However, the histological type distribution, with equal contribution of mucinous and endometrioid types seems to be a unique characteristic of the studied highly miscegenated population.

**Conclusion:**

We have enlighten the profile of the studied ovarian cancer population, which might enable the development of more efficient political strategies to control this malignancy that is the fifth leading cause of cancer-related deaths among women.

## Background

Ovarian tumors can be classified as primary peritoneal carcinoma, Fallopian tube cancer, germinative tumors, benign epithelial ovarian tumors (adenomas), tumors of low malignant potential (borderline tumors), or malignant epithelial tumors (adenocarcinomas); being the latest the focus of the present article. Whereas most epithelial ovarian tumors are benign, do not spread, and usually do not lead to serious illness [1], epithelial ovarian cancer (EOC) is the ninth most common cancer among women, excluding non-melanoma skin cancers, ranking fifth in cancer-related deaths [1]. Indeed, according to the American Cancer Society, EOC accounts for more deaths than any other cancer of the female reproductive system [1]. In the U.S.A., 21.990 new EOC cases, and 15.460 EOC-related deaths are expected in 2011 [1]. The epidemiological scenario of EOC derives, at least partially, from inefficient diagnosis/prognosis strategies mainly due to the lack of specific symptoms at the initial stages of EOC. As a consequence, about 70% EOC are diagnosed at advanced stages when the usually metastatic tumor has acquired drug resistant phenotype [2].

World public health systems are dramatically affected by the inexistence of specific and sensitive EOC biomarkers; therefore compromising the early detection of the disease when patients' survival rates would be as high as 85% [3]. Nonetheless, the two screening tests available for the detection of sporadic EOC - transvaginal sonography and serum CA-125 dosage - have been proven unspecific so that their diagnostic relevance remains controversial. Regarding EOC therapeutics, the standard procedure includes cytoreduction followed by platinum-based adjuvant chemotherapy. Unfortunately, many patients will experience disease recurrence and will ultimately die from EOC [4].

It has been documented that the higher incidence of EOC is among women at their 60's or older [1]. As the world's population ages, remarkable increases in the total number of EOC cases are expected [5], emphasizing the importance of EOC in public health matters. The Brazilian National Institute of Cancer (INCA) describes EOC as a high mortality gynecological malignancy [6]. In spite of the impact of EOC statistics in public economy, Brazil still lacks precise epidemiologic data on the disease, which would support the development of sustainable and more efficient political strategies to control the malignancy.

In conclusion, Brazil urges for epidemiological studies on EOC to characterize and understand the disease profile in specific populations. Herein, we present a pioneer epidemiologic study of ovarian tumors aiming to characterize the disease in Espírito Santo, a Brazilian State with highly miscegenated population, as will be further discussed, regarding the characteristics associated with EOC, such as: patients' age at diagnosis, ovarian neoplasia pathologic profile (histological classification, tumor staging, and tumor degree of differentiation), responsiveness to chemotherapy, and patients' clinical outcome and survival rate.

## Methods

### Data source

A retrospective study conducted with primary ovarian neoplasia (benign or malignant) cases registered in the two reference public hospitals in cancer diagnosis and treatment in the state of Espírito Santo (Brazil): Hospital Universitário Cassiano Antônio de Morais (HUCAM) and Hospital Santa Rita de Cássia (HSRC). Clinical included: patients' age at diagnosis, tumor histological type, tumor FIGO stage, tumor degree of differentiation, chemotherapy regimens, chemotherapy responsiveness or resistance, disease recurrence and disease-free period, and patients' survival. EOC classification has been collected from patients' clinical reports, and has been performed by the Pathology Departments from the referred hospitals, following high laboratorial quality control systems. The present work has been conducted in observation with human rights recommendations, following UFES's Institutional Review Board approval (protocol # 042/07; approval date 01/08/2007); all patients involved have signed the Term of Free and Informed Consent and their clinical follow up information were kept in confidential records.

### Geographic characteristics of the State of Espírito Santo, Brazil

The present data have been collected at the Brazilian State of Espírtito Santo, located in the Southeastern Brazil, which capital is Vitória (-20° 19' 10'' S, 40° 20' 16'' W), comprising a total area of 46,077,519 km^2^. According to Brazilian Institute of Geography and Statistics [7] (Instituto Brasileiro de Geografia e Estatística, IBGE, from Portuguese), the estimated population of the State in 2009 has been 3,487,199 people.

### Cohort definition

The present study included all ovarian adenomas, borderline tumors, and cancers registered at HUCAM from 1997 to 2007 and at HSRC from 2001 to 2007. Despite this extensive sampling, the main focus of the present study was the epidemiological characterization of primary ovarian malignant epithelial tumors. With this regard, we have excluded all cases of non-primary ovarian tumor and non-epithelial EOC from the analysis.

### Statistical Analysis

Data are expressed as absolute values and percentage or as mean ± standard deviation (SD). Statistically relevant differences among age at diagnosis were accessed using Students' T-Test or one-way analysis of variance (ANOVA), followed by Turkey post-test to perform individual comparisons. EOC FIGO stage distribution varying by tumor histological type has been compared using chi-square test. For further analysis, we have grouped tumor classified as FIGO stages I and II as a better prognosis disease group, whereas tumors designated as FIGO stages III and IV as a poor prognosis disease group, and, once again, analyzed its distribution among histological types using chi-square test.

To better understand and characterize our studied population, Kaplan-Meier curves have been plotted using patients' death or disease relapse as endpoints (considered overall survival and disease-free survival, respectively). The curves have been generated for each of the mainly observed histological types (endometrioid, mucinous, and serous), patients' age at diagnosis separated in groups (less than 40 years old, 41 to 60 years old, and 61 or more years old), EOC FIGO stage (I, II, III, and IV), and adjuvant chemotherapy regimen (platinum only, platinum associated with paclitaxel, and platinum associated with cyclophosphamide). Curves have been compared using the Mantel-Cox Log-Rank test. It is important to notice that only patients who underwent one single type of adjuvant therapy have been included in this analysis. Data are expressed as p-value of Log-Rank analysis, harzard ratio (HR), and 95% Confidence Interval (IC95%). All statistical analyses have been performed using GraphPad Prism 5.0 software.

## Results and Discussion

### Results

In the present, we have analyzed 248 primary ovarian epithelial neoplasias: 83 adenomas, 19 borderline tumors and 146 cancers, registered in the two main cancer services of the state of Espírito Santo, Brazil, as described in the Methods section. The population characterization revealed that the mean age of women at ovarian neoplasia diagnosis was 49.86 ± 15.22. Interestingly, there was significant statistic difference of women's age at diagnosis according to the type of ovarian epithelial neoplasia (adenoma, borderline tumor, and cancer) (one way ANOVA p < 0.0001). For EOC, the mean patients' age at diagnosis was 54.67 ± 13.84, which was significantly different from that of borderline tumors (46.15 ± 11.15; Turkey post-test, p < 0.05), and adenomas (42.01 ± 15.06; Turkey post-test, p < 0.0001). No significant difference has been noted between women's age at diagnosis for adenomas and borderline tumors (Table 1). As for FIGO staging, the mean age at diagnosis was 47.97 ± 11.54 for stage I, 51.60 ± 12.50 for stage II, 56.89 ± 14.44 for stage III and 60.27 ± 11.16 for stage IV, showing a possible positive correlation between patients' age at diagnosis and tumor FIGO staging (data not shown). As stated in the Methods section, EOC graded as stages I and II are related to better prognosis disease, whereas stages III e IV correspond to poorest prognosis EOC. We have observed a statically relevant difference between the patients' age at diagnosis between the group of better prognosis (48.90 ± 11.74) and the group of poorest prognosis disease (57.90 ± 13.52; T-Test p = 0.0014; data not shown).

**Table 1:** Characterization of the ovarian tumor cases registered (diagnosed and/or treated) at the collaborator hospitals

Parameters	EOC and stages					Borderline	Adenoma
			
	Total	I	II	III	IV		
N	146	29	10	35	15	19	83
Age at diagnosis (mean ± SD)	54.67 ± 13.84*	47.96 ± 11.54	51.6 ± 12.5	58.9 ± 14.4	60.3 ± 11.2	46.15 ± 11.15	42.01 ± 15.06
30 or less	8 (5.5%)	3 (10.3%)	1 (10,0%)	2 (5.7%)	0 (0.0%)	2 (10.5%)	21 (25.3%)
31 to 40	11 (7.5%)	3 (10.3%)	1 (10,0%)	1 (2.9%)	0 (0.0%)	4 (21.1%)	19 (22.9%)
41 to 50	42 (28.8%)	12 (31.4%)	2 (20,0%)	13 (37.1%)	4 (2.7%)	7 (36.8%)	13 (15.7%)
51 to 60	32 (21.9%)	5 (17.2%)	4 (40,0%)	3 (8.6%)	4 (2.7%)	3 (15.8%)	15 (18.0%)
Over 60	52 (35.6%)	4 (13.8%)	2 (20,0%)	16 (45.7%)	7 (46.7%)	3 (15.8%)	12 (14.5%)
Missing data	1 (0.7%)	0 (0.0%)	0 (0,0%)	0 (0.0%)	0 (0.0%)	0 (0.0%)	3 (3.6%)

* One-way ANOVA: p < 0.05 when compared to borderline and p < 0.001 when compared to adenoma

Pathologic profile of EOC included in this study is presented in Table 2. As expected, we have observed a higher prevalence of ovarian serous adenocarcinoma (n = 44) when compared to ovarian mucinous adenocarcinoma (n = 20), ovarian endometrioid adenocarcinoma (n = 20), and ovarian clear cell adenocarcinoma (n = 3). Considering the most prevalent primary EOC histological types (serous, mucinous, and endometrioid), there was no statistical significant differences at women's age at diagnosis. However, it is worthwhile to point that the only histological type that affected women under the age of 30 year old was serous carcinoma, while for other EOC histological types every case was diagnosed after this age, and this tendency is statistically significant (Fisher's test, p = 0.0126; data not shown) (Table 3).

**Table 2:** Pathologic profile of epithelial ovarian cancers.

Parameters	N (%)
Histological Type	
Serous	44(30.1)
Mucinous	20(13.7)
Endometrioid	20(13.7)
Clear cells	3(2.1)
Adenocarcinoma without other specification	58(39.7)
Others	1(0.7)
FIGO Stage	
I	29(19.9)
II	10(6.9)
III	35(23.9)
IV	15(10.3)
Missing data	57(39.0)
Differentiation Grade	
Well	18(12.3)
Moderate	30(20.6)
Poor	17(11.6)
Missing data	81(55.5)
Laterality	
Left	11(7.5)
Right	12(8.2)
Bilateral	29(19.9)
Missing data	94(64.4)

**Table 3:** Characteristics of the most observed cancer histological types.

Parameters	Histological Type			Totalª
		
	Endometrioid	Serous	Mucinous	
Age at diagnosis (mean ± SD)	54.80 ± 12.76	52.70 ± 15.95	51.76 ± 8.85	52.72 ± 13.71
30 or less	0(0.0%)	7(15.9%)	0(0.0%)	7(8.4%)
31 to 40	2(10.0%)	3(6.8%)	1(5.0%)	6(7.1%)
41 to 50	8(40.0%)	10(22.7%)	11(55.0%)	29(34.5%)
51 to 60	2(10.0%)	9(20.5%)	3(15.0%)	14(16.7%)
More than 60	8(40.0%)	15(34.1%)	5(25.0%)	28(33.3%)
FIGO Stage				
I	7(35.0%)	11(25.0%)	5(25.0%)	23(27.4%)
II	2(10.0%)	6(13.6%)	1(5.0%)	9(10.7%)
III	1(5.0%)	13(29.6%)	7(35.0%)	21(25.0%)
IV	2(10.0%)	2(4.5%)	2(10.0%)	6(7.1%)
Missing data	8(40.0%)	12(27.3%)	5(25.0%)	25(29.8%)

ªTotal cases of EOC specified as endometrioid, serous or mucinous.

Regarding EOC FIGO classification, we have documented 29 cases diagnosed at stage I, 10 cases detected at stage II, 35 cases diagnosed at stage III, and 15 cases detected at stage IV, in a total of 89 staged cancers. We have also observed a slight predominance of poor staged cancers, as 43.8% of cases had a better prognosis profile (I and II) versus 56.2% of the cases that showed a poor prognosis standard (III and IV). Analyzing the FIGO staging of the tumor within each histological type category, we could not establish any statistically relevant association. Interestingly, 58.3% of the ovarian endometrioid adenocarcinomas, for which data was available, were FIGO-classified as stage I tumors, whereas for serous and mucinous ovarian adenocarcinomas the referred proportions were 34.4% and 33.3%, respectively (Table 3). Among EOC with available degree of differentiation data, we have noticed a prevalence of moderately differentiated tumors, while poorly differentiated and highly differentiated ones were observed almost at the same range (Table 2). As for laterality of the tumors, only 35.6% of the analyzed clinical charts documented this disease characteristic, and bilateral EOCs were slightly more frequent than unilateral ones (Table 2).

Patients' clinical outcome and chemotherapy regimens prescribed to EOC patients have been also analyzed, and the correspondent data are compiled in Tables 4 and 5. Considering patients for which data was available only, we have observed that 10% of the patients underwent neoadjuvant therapy, 78% of them received adjuvant therapy and, from those whose disease has relapsed, 92% got post-relapse therapy. Concerning the chemotherapy regimens prescribed to EOC patients, we have noted: i) for neoadjuvant therapy: 90% of the patients received a platinum-based regimen, being 70% of them in association with paclitaxel; ii) for adjuvant therapy: about 82.4% of the patients got the first choice adjuvant therapy with platinum-based drug, being 47.1% treated with platinum associated with paclitaxel, 20.6% platinum associated with cyclophosphamide, and 14.7% platinum alone; iii) for post-relapse therapy: 56.4% of the patients received platinum-based therapy. As for platinum-based therapy responsiveness, 17.1% of the patients were resistant to the treatment, whereas 22.6% were susceptible to this therapy (Table 4).

**Table 4:** Treatment and clinical outcome of the ovarian cancer patients

Parameter	N (%)
Neoadjuvant Therapy	
Yes	10(6.9)
No	91(62.3)
Missing data	45(30.8)
Adjuvant Therapy	
Yes	68(46.6)
No	16(11.0)
Deceased before therapy	3(2.0)
Missing or insufficient data	59(40.4)
Post-Relapse Therapy	
Yes	23(74.2)
No	2(6.3)
Missing or insufficient data	6(19.5)
Clinical Outcome	
Cure	7(4.8)
Disease-free	23(15.7)
Relapse	30(20.5)
Death before relapse	24(16.4)
Relapse after cure	1(0.7)
Missing or insufficient data	61(41.8)
Platinum responsiveness	
Resistant	25(17.1)
Susceptible	33(22.6)
No platinum-based therapy	17(11.6)
Missing or insufficient data	71(48.6)

**Table 5:** Chemotherapy regimens prescribed to the ovarian cancer patients.

Chemotherapy	N (%)
Neoadjuvant	
Platinum	1(10.0)
Platinum + Paclitaxel	7(70.0)
Platinum + Cyclophosphamide	1(10.0)
No information	1(10.0)
Adjuvant	
Platinum	10(14.7)
Paclitaxel	3(4.4)
Cyclophosphamide	1(1.4)
Platinum + Paclitaxel	32(47.1)
Platinum + Cyclophosphamide	14(20.6)
Doxorubicin	1(1.5)
No information	7(10.3)
Post-Relapse	
Platinum	4(17.3)
Paclitaxel	2(8.7)
Gemcitabine	5(21.7)
Topotecam	1(4.4)
Etoposide	1(4.4)
Platinum + Paclitaxel	9(39.1)
No information	1(4.4)

Chemoterapies most commonly applied

**Figure 1:**
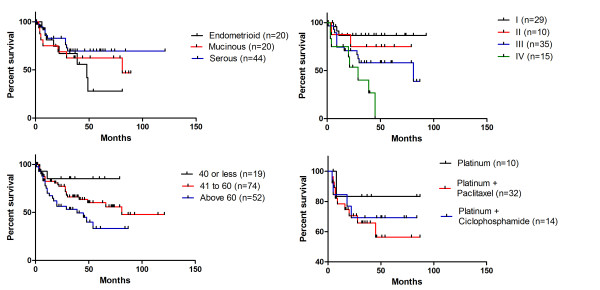
**Kaplan-Meier Overall survival curves**. A: Overall survival comparison among histological types. Endometrioid vs. Mucinous (Log-Rank p = 0.562; HR = 1.31; CI95% = 0.48-3.82); Endometrioid vs. Serous (Log-Rank p = 0.152; HR = 2.12; CI95% = 0.76-5.89); Serous vs. Mucinous (Log-Rank p = 0.421; HR = 1.53; CI95% = 0.54-4.35). B: Overall survival comparison among FIGO stages. I vs. II (Log-Rank p = 0.452; HR = 2.16; CI95% = 0.29-16.28); I vs. III (Log-Rank p = 0.033; HR = 3.07; CI95% = 1.09-8.61); I vs. IV (Log-Rank p = 0.0003; HR = 12.56 CI95% = 3.19-49.46); II vs. III (Log-Rank p = 0.458; HR = 1.63; CI95% = 0.45-5.95); II vs. IV (Log-Rank p = 0.025; HR = 4.40; CI95% = 1.20-16.13); III vs. IV (Log-Rank p = 0.055; HR = 2.87; CI95% = 0.97-8.45). C: Overall survival comparison among groups of age at diagnosis. 40 years old or less vs. 41 to 60 years old (Log-Rank p = 0.170; HR = 2.04; CI95% = 0.73-5.64); 40 years old or less vs. above 60 years old (Log-Rank p = 0.018; HR = 2.91; CI95% = 1.20-7.04); 41 to 60 years old vs. above 60 years old (Log-Rank p = 0.057; HR = 1.817; CI95% = 0.98-3.36). D: Overall survival comparison among adjuvant treatment regimens. Platinum only vs. Platinum-Paclitaxel (Log-Rank p = 0.347; HR = 2.02; CI95% = 0.46-8.80); Platinum only vs. Platinum-Ciclophosphamide (Log-Rank p = 0.499; HR = 1.89; CI95% = 0.30-1.99); Platinum-Paclitaxel vs. Platinum-Ciclophosphamide (Log-Rank p = 0.633; HR = 1.31; CI95% = 0.43-4.00).

Finally, EOC patients' overall survival (OS) profile has been investigated in regard to the disease histological type, FIGO staging, adjuvant therapy, and patients' age at diagnosis (Figure 1). For this analysis, we have considered the most prevalent EOC histological types (serous, endometrioid, and mucinous) of the studied population, and the most common prescribed adjuvant therapies (platinum only, platinum associated with paclitaxel, or platinum associated with cyclophosphamide). We have not been able to correlate histological type or adjuvant therapy with patients' OS (Log-Rank p > 0.05). On the other hand, and configuring crucial information regarding the epidemiological profile of EOC, women's OS was statistically associated with the patients' age at diagnosis (Log-Rank p = 0.029) and the tumor FIGO staging (Log-Rank p = 0.004). Indeed, performing every possible pair of comparisons, we have observed that patients diagnosed with EOC after the age of 60 years old had described a poor OS when compared to the group of patients diagnosed before the age of 40 years old (Log-Rank p = 0.0179), and also when compared to the group of patients diagnosed between 41 and 60 years old (p = 0.057). Although the Kaplan-Meier curve shows a different trend of OS regarding patients diagnosed before 20 years old and patients diagnosed between 41 and 60 years old, no statistically significant association has been observed (Log-Rank p = 0.169). The same type of analysis was performed with FIGO data: patients carrying EOC FIGO-staged IV showed a poor OS when compared to those classified as stage I tumors (Log-Rank p = 0.0003), stage II (Log-Rank p = 0.025), and stage III (p = 0.055). Additionally, the OS difference between patients diagnosed with EOC FIGO-staged I or III was also statistically relevant (Log-Rank p = 0.033). The other pairs of comparisons have not shown any statistically different survival trend (Figure 1).

**Figure 2:**
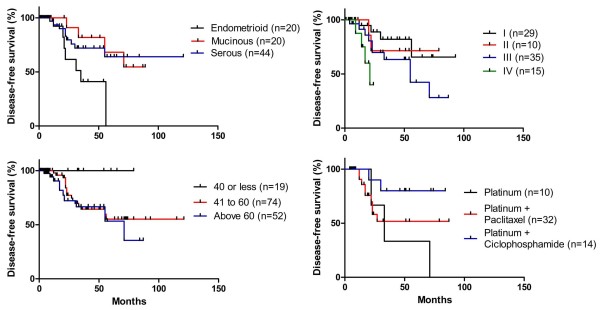
**Kaplan-Meier Disease-free survival curves**. A: Disease-free survival comparison among histological types. Endometrioid vs. Mucinous (Log-Rank p = 0.016; HR = 5.18; CI95% = 1.36-19.74); Endometrioid vs. Serous (Log-Rank p = 0.048; HR = 3.29; CI95% = 1.01-10.70); Serous vs. Mucinous (Log-Rank p = 0.072; HR = 1.19; CI96% = 0.37-3.82). B: Disease-free survival comparison among FIGO stages. I vs. II (Log-Rank p = 0.071; HR = 1.31; CI95% = 0.38-7.87); I vs. III (Log-Rank p = 0.109; HR = 2.38; CI95% = 0.82-6.88); I vs. IV (Log-Rank p = 0.005; HR = 17.09; CI95% = 2.31-126.07); II vs. III (Log-Rank p = 0.408; HR = 1.73; CI95% = 0.47-6.37); II vs. IV (Log-Rank p = 0.136; HR = 3.66; CI95% = 0.66-20.18); III vs. IV (Log-Rank p = 0.157; HR = 3.64; CI95% = 0.75-17.75). C: Disease-free survival comparison among groups of age at diagnosis. 40 years old or less vs. 41 to 60 years old (Log-Rank p = 0.026; HR = 3.75; CI95% = 1.17-11.91); 40 years old or less × above 60 years old (Log-Rank p = 0.021; HR = 4.72; CI95% = 1.26-17.72); 41 to 60 years old × above 60 years old (Log-Rank p = 0.406; HR = 1.42; CI95% = 0.62-3.28). D: Disease-free survival comparison among adjuvant treatment regimens. Platinum only × Platinum-Paclitaxel (Log-Rank p = 0.488; HR = 1.72; CI95% = 0.37-7.95); Platinum only × Platinum-Ciclophosphamide (Log-Rank p = 0.042; HR = 8.95; CI95% = 1.08-73.95); Platinum-Paclitaxel × Platinum-Ciclophosphamide (Log-Rank p = 0.121; HR = 2.65; CI95% = 0.77-9.05).

Kaplan-Meier curves have been also generated using EOC relapse as the endpoint, in order to analyze the disease-free survival (DFS) in regard to the tumor histological type, tumor FIGO staging, patients' age at diagnosis, and adjuvant therapy regimen prescribed to the EOC patient. We had observed a statistically relevant different DFS when comparing FIGO stages (Log-Rank, p = 0.035), but only limitrophe statistical relevance when compared tumor histological types (Log-Rank, p = 0.056) and the patients' age at diagnosis (Log-Rank, p = 0.0529). No statistically relevance could be noted when adjuvant treatments were compared (Log-Rank, p = 0.126). Comparing every possible pair of curves, we had come to some interesting findings. As for tumor histological type, the DFS in endometrioid cancers was lower than in serous (Log-Rank, p = 0.048) and in mucinous (Log-Rank p = 0.016) EOC. Regarding tumor FIGO staging, the only statistically relevant decrease in DFS has been observed between the tumors at stages I and IV (Log-Rank, p = 0.0054). Patients diagnosed with EOC at the age of 40 years old or less have shown a higher DFS comparing to patients whose diagnosis has occurred between the ages of 41 and 60 years old, and also to patients whose cancer has been detected at an age older than 60 years old (Log-Rank, p = 0.026 and p = 0.021, respectively). Although no overall statistically relevant difference has been observed for the type of adjuvant treatment received by EOC patients, the ones treated only with platinum have shown a lower DFS than those treated with the association of platinum and ciclophosphamide (Log-Rank, p = 0.042). Every other comparison has not resulted in statistically relevant results, even though the curves may show some interesting trends (Figure 2).

## Discussion

As the world population ages, governmental health policy might consider the social, economic, and psychological impacts of age-related diseases, as cancer, on individuals quality of life. In developing countries, as Brazil, the referred increased lifespan of the population is a milestone event in determining efficient and sustainable strategies regarding public health matters. Moreover, it has been recently reported that Brazilian women are expected to live longer than men, supporting the urge in establishing gender-specific health guidelines [8]. In this context, it is of fundamental importance to conduct epidemiological studies on female issues, as EOC, to understand and characterize not exclusively the disease profile in specific populations, but also to enable the proposition of more efficient diagnosis and therapeutic approaches. Even though EOC incidence is considerably lower than that of other cancers, as breast cancer, the lack of disease pathognomonic symptoms, specific biomarkers, and efficient diagnosis tests result in its detection at metastatic and late stages when the disease prognosis is poor. Therefore, regardless its ninth position in incidence, it is the fifth leading cause of cancer-related deaths among women [1,9]. Besides the emotional impact in carriers' life, cancer management is associated to high economic cost both to public and private health care systems, including expendures with chemotherapy, health care, side effects control, and lost or decreased ability to work [10]. On the other hand, as estimated by a Brazilian health insurance company, treatment of late-diagnosed cancer can cost eight times more money than the control of early-staged malignancy [6].

The World Health Organization has estimated 224,747 new cases of EOC in the world population in 2008. From these, approximately 43% would be diagnosed in women older than 60 years old [11]. North-American statistics have pointed that women are usually diagnosed with EOC after menopause. Indeed, approximately 50% of EOC cases registered in the USA are diagnosed in women at the age of 60 years old or later [1]. In the present study, we have observed that the mean patients' age at EOC diagnosis was 54.67 ± 13.84, which is in consonance with national and international publications [12-17]. Similarly to what is observed in breast cancer, estrogen and hormone therapy (HT) seems to play an important role in EOC [18]. An increased EOC incidence after combined estrogen plus progestin therapy was suggested by a randomized, double-blind, placebo-controlled trial including 16,608 postmenopausal women [19]. Yet, when we consider the dualistic classification of EOC (further discussed in this section), estrogen shows distinct roles. In type I EOC, it acts as a continued growth stimulus to promote cell proliferation; whereas, in type II EOC, it acts on an initiating event rather than as a growth factor [20]. On the other hand, women are usually diagnosed with ovarian borderline tumors at younger ages. In a study conducted in Singapore, Wong et al [21] have reported that the mean women's age at ovarian borderline tumors diagnosis was 38 years old, ranging from 16-89 years old. Another study developed in France have documented that one third of ovarian borderline tumors are diagnosed in women younger than 40 years old, and more than 80% of cases are detected early in an disease course [22]. Moreover, these tumors tend to affect women at a younger age than the typical EOCs in the USA [1]. Correspondingly, our data have shown that the average age of patients diagnosed with borderline tumors (46.15 ± 11.15) was significantly lower (p <0.05) than the age at diagnosis of EOC cases (54.67 ± 13.84). The tendency to correlate more aggressive tumors diagnosed at late ages is corroborated by the results showing that ovarian adenomas are also diagnosed earlier than EOC [9]. The mean age at ovarian adenoma diagnosis in our studied population was 42.01 ± 15.06, significantly lower (p <0.001) than that observed for EOC detection (54.67 ± 13.84). Therefore, our data substantiate the statement that the malignant transformation of normal cells is an aging-related phenomenon. More specifically, EOC is, in fact, a disease of the aged women.

As reported by other groups, serous carcinoma is the most common histological type of EOC [23-26]. In agreement, our group has identified 44 ovarian serous adenocarcinomas, corresponding to 30.1% of the analyzed cases. Furthermore, some groups found ovarian mucinous adenocarcinoma as the second most frequent diagnosed EOC [17,26], including other studies conducted in Brazil [16,17,27], whereas others have pointed to ovarian endometrioid adenocarcinoma occupying the second position in EOC incidence rank [24,25]. In contrast, our analysis has indicated equal proportions of both ovarian mucinous and endometrioid adenocarcinoma within the EOC cases evaluated (13.7%). Intriguingly, all cases of EOC diagnosed in women younger than age 30 years old were of serous type. Though remarkable, this observation is novel, at least to our knowledge, and has not been described in other epidemiological correlated studies. Even though the referred Brazilian EOC studies did not aim to analyze the epidemiology of the disease, therefore might not be considered an absolute distribution of EOC cases in the populations studied, the lack of correlated data in the literature lead us to compare our results to the fractioned population described in the cited articles. One interesting aspect is that all the Brazilian studies mentioned above were performed in São Paulo State, although in distinct cities, in a way that, altogether, they might enlighten EOC profile in the State as a whole. It has been described that São Paulo State has a distinct population composition when compared to Espírito Santo State. According to IBGE data from 2008 [28], the populations from São Paulo and Espírito Santo States are composed by, respectively: 67.2% and 42.2% Caucasian; 6.2% and 8.5% Afrodescendant; 25.4% and 48.6% Brown (Pardo) and 1.3% and 0.7% Native Brazilians. These data indicate the higher population miscegenation observed in Espírito Santo State, which is explained mainly by several immigration cycles that have began in the 14^th ^Century with the Brazilian colonization by Portugal. During the following years, the Espírito Santo State has also received immigrants from Africa, Italy, Spain and Germany, as well as from other regions from Brazil [29]. Finally, as published in 2008 by IBGE [28], the State of Espírito Santo has a higher percentage of miscegenated population (Afrodescendant, Caucasian and Native Brazilians) when compared to the overall Brazilian population (50.6% and 42.6%, respectively).

Most of the EOC cases analyzed herein were FIGO-staged as I or III tumors (19.9% and 23.9%, respectively), in agreement with data published by Kim et al [30], who have reported an incidence of 39% of EOC detected in stage I, and 42.7% of EOC diagnosed in stage III in a study performed in Chicago, USA. In another study conducted in USA, the authors have observed the same disease pattern [31]. The EOC FIGO-staging profile has also been documented in other studies performed in Brazil: Badiglian Filho et al [27] have reported an incidence of 26.3% and 42%, and Derchain et al [32] of 34% and 51%, of EOC staged I and III, respectively. Interestingly, our data have shown a slightly higher predominance of poor prognosis EOC (stages III and IV), which accounted for 56.2% of the cases, while 43.8% of the tumors were in the group of better prognosis disease (stages I and II), when only the cases containing information are considered. Our data is in accordance with other studies conducted in the USA, Indonesia, and Brazil [26,27,32,33].

Expanding our EOC epidemiological analysis to the patients' OS profile, our group has not observed significant differences in death risk among EOC histological types. Nevertheless, ovarian endometrioid adenocarcinomas have a lower DFS than any other EOC histological types. In contrast, others have pointed to the ovarian mucinous adenocarcinomas as the poorest prognosis EOC, which have a lower progression-free survival when compared to ovarian endometrioid and serous adenocarcinomas [14,34]. Although we cannot explain the differences between our observations and that of other authors, one might speculate that lower DFS identified within the ovarian endometrioid adenocarcinomas group could be due to the higher patients' age at EOC diagnosis for this histological type in the studied population.

EOC therapeutic management is based on the combination of a platinum-derived compound (carboplatin, mainly, or cisplatin), and a taxane (paclitaxel). As documented by Aebi and Castiglione [35], the referred drug combination seems to confer a better response rate than the platinum-derived compound alone, therefore increasing EOC carriers' survival rate. In agreement with worldwide EOC therapeutic guidelines, the association of platinum-derived drug and paclitaxel was the first choice chemotherapy regimen prescribed to the investigated EOC patients in both neoadjuvant and adjuvant schemes. The cited drug association has been given to 70% and 47.1% of EOC cases, respectively, followed by platinum combined to cyclophosphamide (10% and 20.6%, respectively), or only platinum (10% and 14.7%, respectively). Contrasting to the observations of Aebi and Castiglione [35], we have not identified significant differences in the patients' OS rate or in the DFS in the group of women who have received platinum and paclitaxel compared to those treated with platinum alone or platinum combined to cyclophosphamide. This finding is intriguing as it provides evidences that the control of EOC might be conducted in an efficient but yet less toxic therapeutic regimen, as apart from the classic antineoplasic drugs side effects, paclitaxel is neurotoxic and cyclophosphamide can lead to the development of hemorrhagic cystitis. It is important to emphasize that we are not neglecting the importance of drug combination to control EOC; however, it might be interesting to revisit the standard clinical protocols in a way to increase the disease treatment success in the cases of platinum-resistant EOC, which can account to as much as 80% of all treated EOC in Brazil (unpublished data).

Considering the lack of EOC epidemiology information in Brazil, we strongly believe to have provided substantiated data on the matter. Indeed, results presented herein have enlightened the EOC epidemiology in Espírito Santo State, and due to the State miscegenated population could, in some extent, give broader hints of the disease profile. On the other hand, we have faced some difficulties during the elaboration of this manuscript that might not be neglected. First, despite the highly qualified medical staff involved in the disease diagnosis and patient care, as well as the strict methods and high quality procedures followed by them, we have noticed an insufficient data recording at the patient's medical reports. Second, patients' follow-up has not always been adequate, being shorter than the ideal, and, once more, there has been lack of some information during this period. Finally, new insights related to the development of EOC should be considered during tumor diagnosis and pathologic classification, such as the possible origin of high grade ovarian carcinomas from fallopian tubes and the dualistic model of EOC classification. Classically, the origin of EOC has been referred as from mesothelial cells in which metaplasic changes would lead to different EOC's histological types. However, more recently, it has been proposed that the majority of what seemed to be primary EOC are derived from other organs, such as the Fallopian tubes [36,37]. In this context, Shi and Kurman [38] proposed a dualistic model that categorizes the types of EOC into two groups, designated type I and type II. According to this model, type I comprises tumors confined to the ovary that develops from well established precursors, the borderline tumors; whereas type II is composed of tumors that are aggressive, present in advanced stage, and develops from intraepithelial carcinomas in the fallopian tube. Despite the evidences that EOC classification methods should be revisited, it is of remarkable importance to emphasize that EOC analysis in the Brazilian Public Health Systems, and in most of the private hospitals as well, remains based on the FIGO classification system.

## Conclusions

We herein present a pioneer detailed epidemiological study on EOC, considering the disease pathology aspects, the chemotherapy regimens prescribed to carrier women, and the patients' survival profile. We have corroborated to the statement that the malignant transformation of ovarian normal cells is an aging-related phenomenon, affecting mostly menopausal women. Moreover, EOC cases registered in the highly miscegenated population of the state of Espírito Santo, Brazil, are: i) mainly of the serous histological type followed equally by the mucinous and endometrioid types; ii) of the serous type in all cases diagnosed in women younger than age 30 years old; iii) with lower DFS if classified as endometrioid adenocarcinoma; iv) mostly diagnosed as poor prognosis disease, although still at a lower prevalence than in other Brazilian states; v) equally responsive to the association of platinum and paclitaxel, of platinum and cyclophosphamide or to platinum alone, therefore suggesting that the control of EOC might be conducted in an efficient but yet less toxic therapeutic regimen, and pointing to the need to revisit the standard clinical protocols in a way to increase the disease treatment success in the cases of platinum-resistant EOC. In conclusion, we have characterized the clinicopathological and prognostic aspects of the studied EOC population. We strongly suggest that our data might guide the development of sustainable and more efficient political strategies to improve the control of this malignancy that is the fifth leading cause of cancer-related deaths among woman.

## List of Abbreviations

ACS: (American Cancer Society); ANOVA: (Analysis of variance); CA-125: (Cancer Antigen 125); CI95%: (95% Confidence Interval); DFS: (Disease-free survival); EOC: (Epithelial Ovarian Cancer); FIGO: (International Federation of Gynecology and Obstetrics); HR: (Hazard Ratio); HSRC: (Santa Rita de Cássia Hospital); HUCAM: (Cassiano Antônio de Moraes University Hospital); INCA: (Brazilian National Institute of Cancer); OS: (Overall survival); SD: (Standard deviation).

## Competing interests

The authors declare that they have no competing interests.

## Authors' contributions

MFP: clinical data collection, manuscript writing, manuscript revision and corrections; RDD, KPM, LCDR, GMS, ALH, LSS, LBCDS, MFC, ACNC: clinical data collection and manuscript writing; AAC: Clinical data collection; IVS: Clinical data collection and paper writing supervision; LBAR: Intellectual mentorship, clinical data collection and paper writing supervision. All authors have read and approved the final manuscript.
